# Holoclone Forming Cells from Pancreatic Cancer Cells Enrich Tumor Initiating Cells and Represent a Novel Model for Study of Cancer Stem Cells

**DOI:** 10.1371/journal.pone.0023383

**Published:** 2011-08-03

**Authors:** Lei Tan, Xin Sui, Hongkui Deng, Mingxiao Ding

**Affiliations:** College of Life Sciences, Peking University, Beijing, China; Technische Universität München, Germany

## Abstract

**Background:**

Pancreatic cancer is one of the direct causes of cancer-related death. High level of chemoresistance is one of the major obstacles of clinical treatment. In recent years, cancer stem cells have been widely identified and indicated as the origin of chemoresistance in multi-types of solid tumors. Increasing evidences suggest that cancer stem cells reside in the cells capable of forming holoclones continuously. However, in pancreatic cancer, holoclone-forming cells have not been characterized yet. Therefore, the goal of our present study was to indentify the holoclone-forming pancreatic cancer stem cells and develop an in vitro continuous colony formation system, which will greatly facilitate the study of pancreatic cancer stem cells.

**Methodology/Principal Findings:**

Pancreatic cancer cell line BxPC3 was submitted to monoclonal cultivation to generate colonies. Based on the morphologies, colonies were classified and analyzed for their capacities of secondary colony formation, long-term survival i*n vitro*, tumor formation *in vivo*, and drug resistance. Flowcytometry and quantitative RT-PCR were performed to detect the expression level of cancer stem cells associated cell surface markers, regulatory genes and microRNAs in distinct types of colonies. Three types of colonies with distinct morphologies were identified and termed as holo-, mero-, and paraclones, in which only holoclones generated descendant colonies of all three types in further passages. Compared to mero- and paraclones, holoclones possessed higher capacities of long-term survival, tumor initiation, and chemoresistance. The preferential expression of cancer stem cells related marker (CXCR4), regulatory genes (BMI1, GLI1, and GLI2) and microRNAs (miR-214, miR-21, miR-221, miR-222 and miR-155) in holoclones were also highlighted.

**Conclusions/Significance:**

Our results indicate that the pancreatic tumor-initiating cells with high level of chemoresistance were enriched in holoclones derived from BxPC3 cell line. Generation of holoclones can serve as a novel model for studying cancer stem cells, and attribute to developing new anti-cancer drugs.

## Introduction

Pancreatic cancer is currently the fourth leading cause of cancer-related mortality. Less than 5% patients survive for 5 years after diagnosis with the median survival period of 4 to 6 months [1], [2]. Although surgical resection is regarded as the most effective method of therapy, its feasibility remains low because of local advancement and early metastasis [3]. In addition, chemotherapy is considered as an important option in clinical therapy, but it usually produces poor effects [4], [5].Therefore, it is necessary to decipher the mechanisms underlying the high level chemoresistance of pancreatic cancer cells.

In recent years, cancer stem cells (or termed as tumor initiating cells) have been identified as an integral part in multi types of solid tumors [6]–[11]. Cancer stem cells not only result in tumor initiation and growth, but also act as the origin of cancer metastasis, relapse and resistance against chemo- or radiotherapy [12]–[14]. Hence, further work on detection and elimination of pancreatic cancer stem cells is still desired to successfully conquer the obstacles in clinical therapy.

Recent studies on adult and cancer stem cells have correlated stem cell properties with the morphology of colonies generated from single cells [15]–[20]. According to the criteria of colony size and borderline defined by pioneering works in keratinocyte cell lines, the colonies were classified and termed as holoclones, meroclones, and paraclones [21]. Similar to stem cells in hair follicles [15], ocular [16], and epidermal [17], cancer stem cells of prostate [19] and glioma [20] were demonstrated to reside in holoclones. Holoclones derived from prostate cancer cell line PC3 initiated tumor formation in NOD/SCID mice exclusively and proliferated in vitro robustly [19]. Cells in holoclones derived from glioma cell line U251 were able to generate tumor spheres in serum free condition and differentiate into lineages of neurons, astrocytes and oligodendrocytes [20]. Interestingly, stem cell related genes were highly expressed in holoclones derived from both prostate cancer and glioma cell lines [19], [20]. These clues suggested that propagation of holoclones from cancer cell lines could serve as an alternative strategy for enrichment of cancer stem cells [20]. However, in pancreatic cancer, holoclones have not been identified and its correlation with properties of cancer stem cells has not been determined yet.

In the present study, we addressed the heterogeneity in pancreatic cancer cell lines BxPC3 [22] and PC3 [23] based on the morphology of colonies derived from single cancer cells and demonstrated that cancer stem cell properties were enriched in holoclones exclusively. Furthermore, our work indicated the holoclone forming cells attribute to chemoresistance, which indicated its potential value to develop chemotherapeutic drugs.

## Results

### Pancreatic cancer cells exhibit heterogeneous capacity to generate diverse colony morphologies in clonal culture

**Figure 1:**
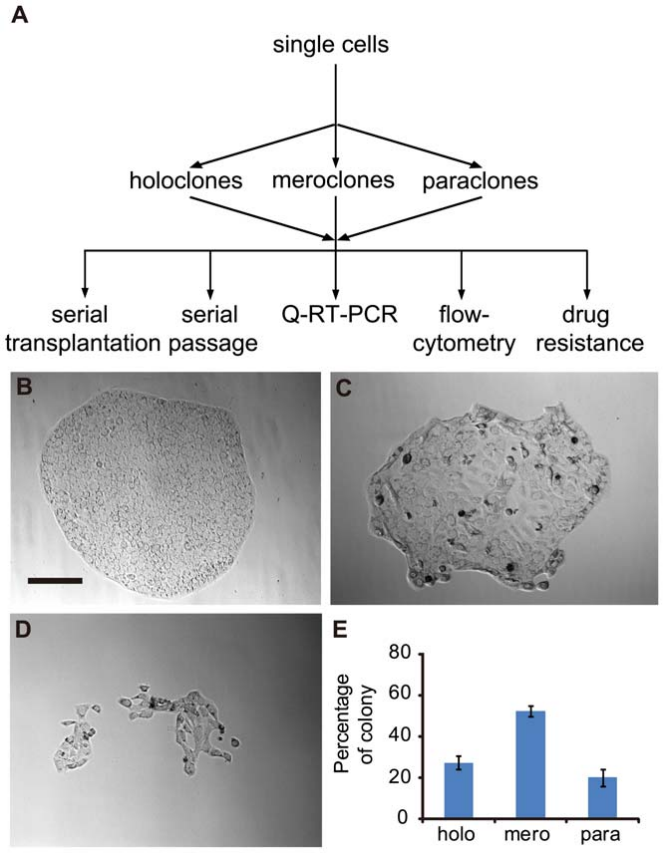
Colony heterogeneity in pancreatic cancer cell line BxPC3. (A) Schematic depicting the procedure of deriving BxPC3 cell clonal cultures and functional assays. Lower panels show representative holoclones (B), meroclones (C) and paraclones (D) from BxPC3 cultures. All photographs were taken at 2 weeks after plating (Bar, 100 microns). At this time point, each type of colonies was counted (E). The results from repeated experiments (n = 4) are presented as means± s.e.m in histogram.

The first aim of our study was to determine whether the diversity of clonal morphologies exists in pancreatic cancer cell population, so monoclonal cultivation was carried out (Fig. 1A) with pancreatic cancer cell line BxPC3. This cell line was derived from primary loci of pancreatic adenocarcinoma and with typical epithelium morphology. After plated, a portion of cells died, while the others were kept viable and formed colonies within 3 days after plating and showed a spectrum of distinguishable morphologies after 5–7 days. Based on differences in morphology, colonies were defined as holoclones, meroclones and paraclones (Fig. 1B, C, D) [21]. Holoclones were clusters of homogeneous, small and tightly packed cells with regular and smooth colony borderlines (Fig. 1B). Paraclones consisted of dispersed and larger cells with fragmented borderlines (Fig. 1D). Meroclones exhibited intermediate morphologies (Fig. 1C). These mophologies were maintained when size of colonies increased. With parallel assays performed with PC3 cell line, three types of colonies were identified (Fig. S1A, B, C) with the morphologies similar to those derived from BxPC3 cell line. The colony composition was similar in these two cell lines: nearly half of the colonies were meroclones, whereas holoclones and paraclones accounted for about 20–30% of the colonies formed (Fig. 1E, Fig. S1D). Therefore, these data indicated the diversity of clonal morphologies in pancreatic cancer cell population.

### Different types of colonies possess differential capacities for self-renewal and long-term proliferation

**Figure 2:**
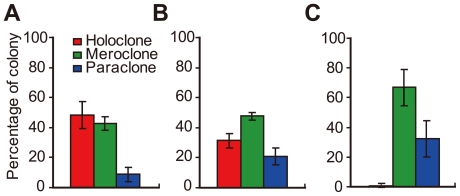
Self-renewal capacity of distinct types of colonies. Cells isolated from single colonies were plated at low density under common condition. (A) At the initial passage, holoclones (n = 8) mainly produced similar frequencies of descendant holoclones and meroclones, whereas much lower percentages of paraclones were generated. (B)After passages of one more month, holoclones (n = 8) generated the full range of progeny colonies at frequencies similar to those retained in unsorted parental cell lines. (C) Meroclones (n = 8) mainly produced paraclones and meroclones, and few holoclones were generated.

**Figure 3:**
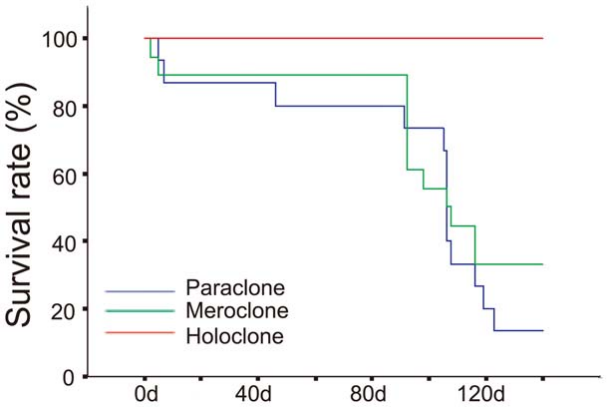
Long-term propagation capacity of distinct types of colonies. Holoclones (n = 12, red line), meroclones (n = 18, green line) and paraclones (n = 15, blue line) were cultured in common condition for 20 weeks and life spans of each colony were recorded. Most of the meroclones and paraclones were aborted gradually, whereas all the holoclones remained viable (p<0.01).

Since the clonal morphological diversity in pancreatic cancer cells had been indicated, the secondary colony formation capacity should be assessed. For this purpose, cells from all three types of colonies were isolated and replated with low density (less than 200 cells per well of 6-well plate) for several passages. At initial passage, the holoclones generated similar proportions of secondary holoclones and meroclones (nearly 50% each), with limited number (less than 10%) of paraclones (Fig. 2A, Fig. S2A). In parallel assays, cells isolated from meroclones produced a rare number of secondary holoclones but a much higher percentage of paraclones (Fig. 2C, Fig. S2C). However, rare cells of paraclones were kept viable after re-plating and generated only secondary paraclones at low frequency (data not shown). To follow the developmental fate of colonies, typical colonies of distinct type were selected for long-term culture. Cells in selected colonies were passaged routinely at clonal density under common condition. For colonies derived from BxPC3 cell line, all 12 holoclones were viable and proliferated robustly, whereas 12 of 18 meroclones and 13 of 15 paraclones were gradually aborted (Fig. 3) during parallel culture of 140 days. Similarly, during 60 days' passage of colonies derived from PC3 cell line, 8 of 9 holoclones were in robust expansion while 4 of 8 meroclones and 6 of 8 paraclones declined in short period (Fig. S3). Interestingly, only cells derived from holoclones regenerated the full range of colonel morphologic phenotypes and restored the proportions of each type of colonies (Fig. 2B, Fig. S2B) similar to the proportions observed in unsorted parental cell lines under low density culture. Based on the distinct appearance exhibited above, the capacity of long-term self-renewal *in vitro* mainly resided in holoclones, but not meroclones or paraclones.

### Holoclones, but not meroclones or paraclones, initiate tumor formation and support tumor serial transplantation in NOD/SCID mice

For the robustness of holoclones had been shown *in vitro*, it was important to evaluate the *in vivo* tumorgenecity of three types of colonies. In order to estimate the tumor formation capacity of each type of colony, serial transplantation assays were performed. Firstly, unsorted BxPC3 cells initiated tumor formation in a dose dependent manner. 100% of mice injected with 10^6^ or 10^5^ BxPC3 cells developed xenograft tumors after 14 days, and mice injected with 10^4^ or 10^3^ cells also developed xenograft tumors after 21 days with 100% efficiency (Table 1). After that, three holoclones, three meroclones, and two paraclones were picked out for transplantation. 10^4^ holoclone cells formed palpable tumors in 100% of mice (15 of 15 mice) within 18 days. On the contrary, no visible tumors were formed by cells from mero- or paraclones (0 of 36 mice, 10^4^∼10^5^ cells per mouse) within 2 months (Table 1). In a further step, cells in xenograft tumors derived from holoclones were then purified and re-transplanted to NOD/SCID mice (10^4^ cells per mouse). Within 18 days, all recipient mice (18 of 18 mice) developed palpable tumors (Table 1). Serial transplantation assays were also performed on PC3 cell line. Unsorted parental cell line, 3 holoclones, 2 meroclones, and 2 paraclones were employed. All the holoclones derived from PC3 cell line were able to develop tumor exclusively in short latency (Table S3).

**Table 1:** Tumorgenecity of distinct types of colonies.

Types of colonies	Clone	Cell number	Tumor incidence	Mean weight of tumor (g)	Termination/latency (day)
Unsorted		10^6^	100% (3/3)		30/14
		10^5^	100% (3/3)		30/14
		10^4^	100% (3/3)		30/21
		10^3^	100% (3/3)		30/21
Para	E03	10^4^	0% (0/6)		60
	E03	10^5^	0% (0/6)		60
mero	E04	10^4^	0% (0/6)		60
	E04	10^5^	0% (0/6)		60
	C02	10^4^	0% (0/6)		60
	C02	10^5^	0% (0/6)		60
Holo	C07 1°	10^5^	100% (5/5)	2.33	48/17
	C07 2°	10^4^	100% (6/6)	0.4	30/15
	D11 1°	10^5^	100% (5/5)	2.49	48/16
	D11 2°	10^4^	100% (6/6)	0.5	30/17
	D10 1°	10^5^	100% (5/5)	3.93	48/14
	D10 2°	10^4^	100% (6/6)	0.69	30/16

NOTE: Cells from BxPC3 clones (1°) at the indicated numbers were injected subcutaneously with 1∶1 mixture of RPMI-1640 and Matrigel into the dorsolateral part of NOD/SCID mice. Secondary (2°) transplantation experiments were carried out as described in “Materials and Methods”.

Exnograft tumors derived from unsorted BxPC3 cell line and corresponding holoclones were analyzed with H&E staining. The histological characteristics of xenograft tumor speciments was visualized and showed high level of similarity between tumor samples from unsorted cell line (Fig. S4A, B) and corresponding holoclones (Fig. S4C, D).

With the significant difference of tumor formation capacity among distict types of colonies, it was suggested that the tumor-initiation capacity *in vivo* was enriched in holoclones rather than meroclones or paraclones.

### Cancer stem cells related surface markers, genes and microRNAs are differentially expressed in distinct types of colonies

**Figure 4:**
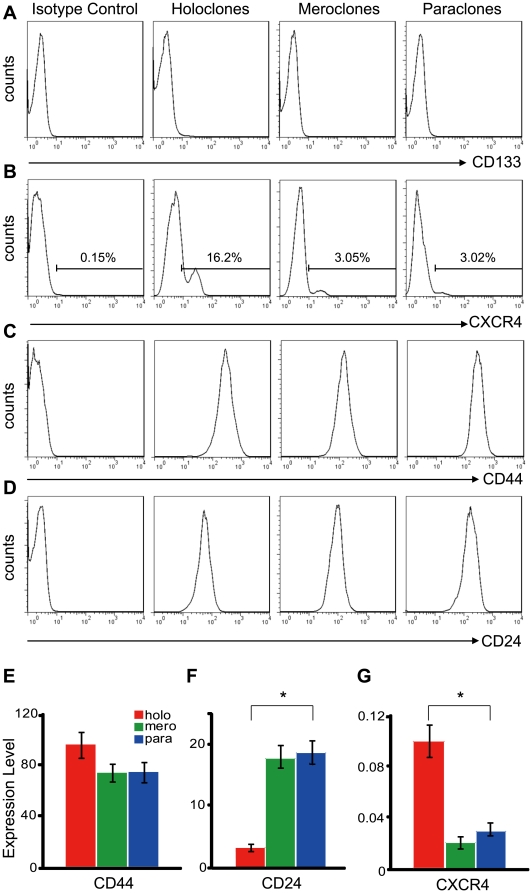
Expression of cancer stem cell markers in holoclones, meroclones and paraclones. Cells isolated from holoclones, meroclones and paraclones were examined with flowcytometry for the cell surface markers of cancer stem cells. CD133 (A) was totally negative in all three types of colonies. The CXCR4^+^ cells (B) were detectable in all types of colonies but significantly enriched in holoclones. CD44 (C) was strongly positive and with little difference among distinct types of colonies, while CD24 (D) was expressed at a higher level in paraclones than in holoclones. mRNA level of *CD44* (E), *CD24* (F) and *CXCR4*(G) in holo-, mero-, and paraclones was also quantified with real-time PCR (*GAPDH* as the internal reference) and similar trends were showed (*, p<0.05).

**Figure 5:**
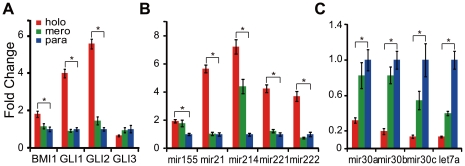
Differential expression of regulatory genes and microRNAs associated with cancer stem cells. *BMI1*, *GLI1*, and *GLI2* were up-regulated in holoclones, while expression of *GLI3* showed no significant change among distinct types of colonies (A). The microRNAs were clustered into two groups, which were elevated (B) and repressed (C) in holoclones respectively. All expression values in distinct types of colonies were normalized against those in paraclones (*, p<0.05).

Based on the characteristics of holoclones *in vitro* and *in vivo*, it was necessary to analysis the expression of cell-surface markers and regulators associated with cancer stem cells in three types of colonies. Therefore, flow-cytometry and quantitative RT-PCR were employed simultaneously. Flow-cytometric assays showed that CD133 was negative (Fig. 4A) and CD44 was positive (Fig. 4C) in holoclones, meroclones and paraclones. All types of colonies contained both CXCR4^+^ and CXCR4^−^ cells, however, the percents of CXCR4^+^ cells were much higher in holoclones than in meroclones and paraclones (Fig. 4B). The CD24 intensity (Fig. 4D, Fig. S5) was significantly stronger in paraclones than in holoclones. Similar results were obtained with quantitative RT-PCR. *CD133* was not detectable and *CD44* showed less than 1.3-fold of up-regulation in holoclones than in paraclones (Fig. 4E). However, expression of *CD24* in holoclones was down-regulated for above 5-fold than in paraclones (Fig. 4F). Expression level of *CXCR4* was about 3-fold higher in holoclones than in paraclones (Fig. 4G). Furthermore, Expression of *BMI-1*, *GLI1*, *GLI2*, *GLI3* and a list of cancer related microRNAs were also quantified. *BMI-1*, *GLI1* and *GLI2* were up-regulated in holoclones rather than in paraclones, while expression level of *GLI3* was not significantly changed among three types of colonies (Fig. 5A). MicroRNAs were showed differentially expressed and clustered into two groups: one group was up-regulated in holoclones, including *miR-214*, *miR-21*, *miR-221*, *miR-222*, and *miR-155* (Fig. 5B); the other group was down-regulated in holoclones, including *Let-7a* and *miR-30c*, *miR-30b*, *miR-30a* (Fig. 5C). The differential expression of markers and regulators also suggest the tendency that stem cell properties were possessed by holoclones rather than other two types of colonies.

### Holoclones exhibit much higher chemoresistance than meroclones and paraclones

**Figure 6:**
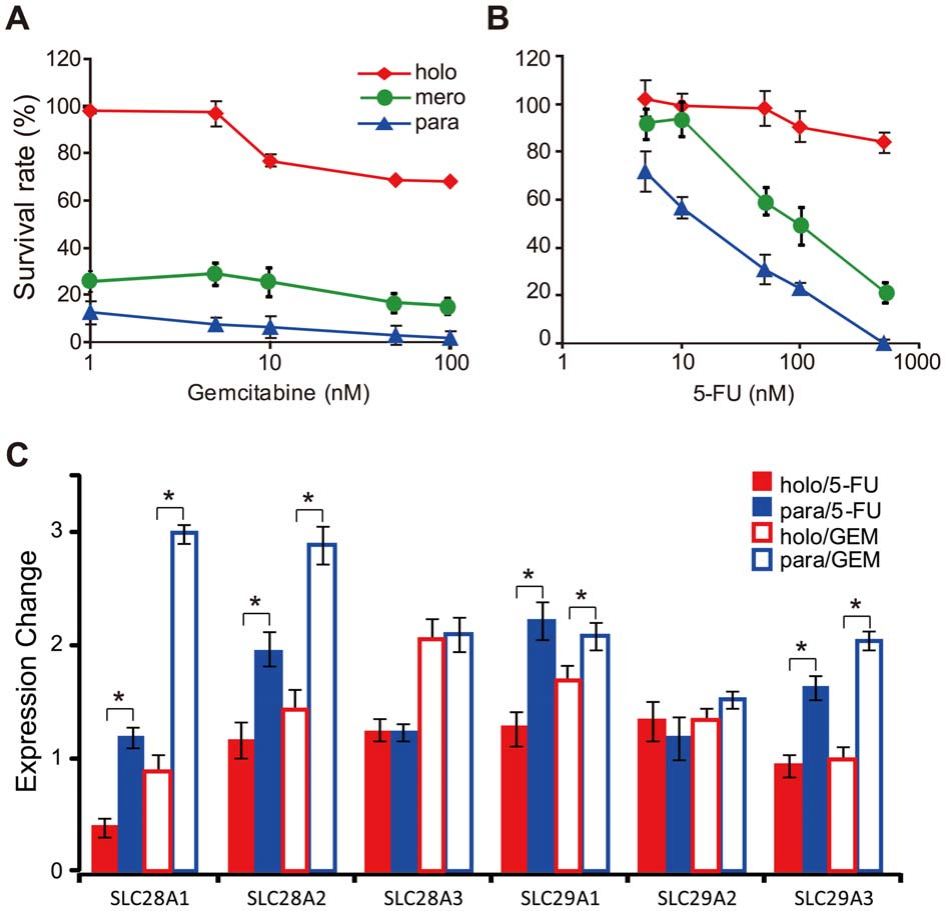
Differences in chemoresistance among holoclones, meroclones and paraclones. Cells isolated from holoclones, meroclones and paraclones were treated with chemotherapeutic drugs of increasing concentration: 1, 5, 10, 50, 100 nM of gemcitabine (A) or 5, 10, 50, 100, 500 nM of 5-FU (B). For each concentration value, three wells were set as repeat in a single experiment and three times of representative experiments were performed. The expression changes of chemo-drug intake transporters were quantified in holoclones and paraclones respectively after treated with 50 nM chemo-drug for 12 hours. (C) (*, p<0.05).

It's well known that chemoresistance is one of the major properties of cancer stem cells, so here we asked whether the holocoloes possess this ability. Hence, the cells isolated from these three types of colonies and treated with gemcitabine and 5-FU under increasing concentration. Among the entire concentration range tested, the survival rates of cells derived from holoclones were significantly higher than those from meroclones and paraclones (Fig. 6A, B). The IC_50_ value of 5-FU was 2.59×10^3^ nM in holoclones, which was much higher than those of meroclones (1.24×10^2^ nM) and paraclones (15.10 nM). To analyze the gene expression change induced by drug treatment, quantitative RT-PCR was carried out with the cell treated with drugs. After treatment with 50 nM of 5-FU for 12 hours, expression of the drug-intake transporters (*SLC28A1*, *SLC28A2*, *SLC28A3*, *SLC29A1*, *SLC29A2*, *SLC29A3*) were all up-regulated in paraclones with no effect in holoclones mostly (only *SLC28A1* was down-regulated about 2-fold) (Fig. 6C).With the parallel treatment of gemcitabine, similar responses of these genes were detected (Fig. 6C). Taken together, *SLC28A1/A2* and *SLC29A1/A3* were commonly up-regulated more dramatically in paraclones than in holoclones after treatments of gemcitabine or 5-FU. This differential response might be, at least partly, invovled in the variation of chemoresistance among three types of colonies.

## Discussion

In present study, we demonstrated the stem cell properties of holoclones and indicated that a panel of stem cell associated genes and microRNAs were preferentially expressed in holoclones. Moreover, we revealed a high level chemoresistance in holoclones and suggested the potential value of holoclones in study of cancer stem cells.

We are the first to show the heterogeneity in clonoal morphologies of pancreatic cancer cells. Similar to the behavior of keratinocyte [21] and multiple cancer cell lines [18]–[20], when cells of BxPC3 and PC3 cell line were plated monoclonally, a serial of colonies with diverse morphologies were developed (Fig. 1A). Based on the morphological diversity, holoclones (Fig. 1B, Fig. S1A), meroclones (Fig. 1C, Fig. S1B) and parclones (Fig. 1D, Fig. S1C) can be easily identified.

Furthermore, our results indicated that stem-like cancer cells were enriched in holoclones rather than mero- or paraclones. During *in vitro* propagation, cells in holoclones generated a high percentage of progeny holoclones at the first round of passage (Fig. 2A, Fig. S2A). After more passages, cells in holoclones generated colonies with full-range of morphological characteristics similar to that derived from unsorted parental cell lines (Fig. 2B, Fig. S2B). However, meroclones generate a limited level of holoclones and much higher percentages of paraclones (Fig. 2C, Fig. S2C). During long-term of passages *in vitro*, holoclones showed more robustness and constant proliferation, while mero- and paraclones declined rapidly (Fig. 3, Fig. S3). With the differences shown above, distinct types of colonies possessed differential capacity of self-renewal and proliferation capacity. More importantly, holoclones serially initiated tumor development *in vivo* while mero- and paraclones did not (Table 1, Table S3), which is regarded as the widely used golden standard for identification of cancer stem cells. As an indispensable supplement, the xenograft tumors derived from BxPC3 holoclones showed similar histological characteristics with those tumor tissue derived from unsorted BxPC3 cell line (Fig. S4). In prostate cancer cell lines PC3 [19] and DU145 [24], similar characteristics of holoclones *in vitro* and *in vivo* had been indicated and served as evidences to support the stem cell property of holoclones.

Moreover, expression of cell surface markers, genes and microRNAs among distinct types of colonies also suggested the stem cell property of holoclones. Firstly, the cell surface markers of pancreatic cancer stem cells, CD44, CD24, and CD133, were evaluated. Based on two independent reports, pancreatic cancer stem cells were identified as population of CD44^+^CD24^+^ESA^+^ or CD133^+^, however, these two populations showed little overlap with each other [10], [11]. According to the results of flowcytometry (Fig. 4A, C) and quantitative RT-PCR (Fig. 4E) assays, expression of *CD133* (undetectable) and *CD44*(highly expressed) were both undistinguishable among three types of colonies. The expression of *CD24* was downregulated in holoclones than in paraclones (Fig. 4F, Fig. S5), which is potentially varied from former report [10]. Similar to the unexpected expression status of *CD133* [11], [25] and *CD24* [10] in pancreatic cell lines BxPC3 and PC3, divergent expression of *CD133* in one certain cell line was also reported in ovarian cancer cell lines OVCAR3 and SKOV3 [26]–[28], which might at least partly due to some undetermined changes induced during long-term cultivation *in vitro*. Moreover, CD133- cancer stem cells have been identified in glioma and suggested as more primordial driven force of tumor development than CD133+ cell population [29]. For further assessment of the stem cell properties of holoclones, a series of genes and mciroRNAs associated with cancer stem cells were quantified. Among the genes up-regulated in holoclones, *BMI1* (Fig. 5A) has been shown to play key regulatory role in self-renewal of neural [30], mammary [31], hematopoietic [32] stem cells and multi types of cancer stem cells [31], [33], [34]. What's more, *BMI1* promoter has been utilized to drive EGFP as an intracellular marker to enrich hematopoietic stem cells [35]. Similar to our results, *BMI1* had been recently reported preferentially expressed in holoclones derived from glioma cell line [20]. The elevation of *GLI1* and *GLI2* in holoclones (Fig. 5A) suggests higher activity of Hedgehog signaling, which was consistent with the higher level of *SHH* expression in CD44^+^CD24^+^ESA^+^ cancer stem cells isolated from human primary pancreatic cancer specimens [20]. The Hedgehog signal pathway also plays an essential role in maintaining cancer stem cells in mammary [31] and brain [36]. *CXCR4*, the pivotal mediator of metastasis, was up-regulated in holoclones (Fig. 4B, G) too. In CD133^+^ pancreatic cancer stem cells, the CXCR4^+^ subpopulation is more invasive than autologous CXCR4^−^ subpopulation [11]. Among microRNAs up-regulated in holoclones, *miR-214*, *miR-221*, *miR-222* and *miR-155* (Fig. 5B) were commonly overexpressed in breast cancer stem cells [37]. However, *Let-7a* (Fig. 5C), which was significantly down-regulated in holoclones, plays a negative role in self-revewal, tumorigenicity, and chemoresistance of breast cancer stem cells [12]. Similarly, miR-30a/b/c (Fig. 5C) were also overexpressed in paraclones. In breast cancer, this microRNA family inhibits self-renew of stem cells, induces apoptosis, and reduces the metastasis to lung [38].

Higher level of chemoresistance was indicated in holoclones rather than in meroclones and paraclones (Fig. 6A, B), which is consistent with the supportive role of cancer stem cells in chemoresistance reported previously [11], [13]. In accordance with the robust chemoresistance in holoclones, genes and microRNAs that sustain chemoresistance, including *BMI1* [39], *GLI1/2* [40], *CXCR4* [41], *miR-214* [42], *miR-21* [43], and *mir-155* [44] (Fig. 4G, Fig. 5A, B) were up-regulated in holoclones. In response to drug treatments, the expression of drug-intake transporters, of which the higher expression level was correlated with longer survival of patients [45]–[48], was induced in paraclones preferentially (Fig. 6C). This means the drug in-take will be increased more rapidly in paraclones than in holoclones. This could be one of the potential origins of preferential survival of cancer stem cells versus non-stem cancer cells in chemotherapy.

**Figure 7:**
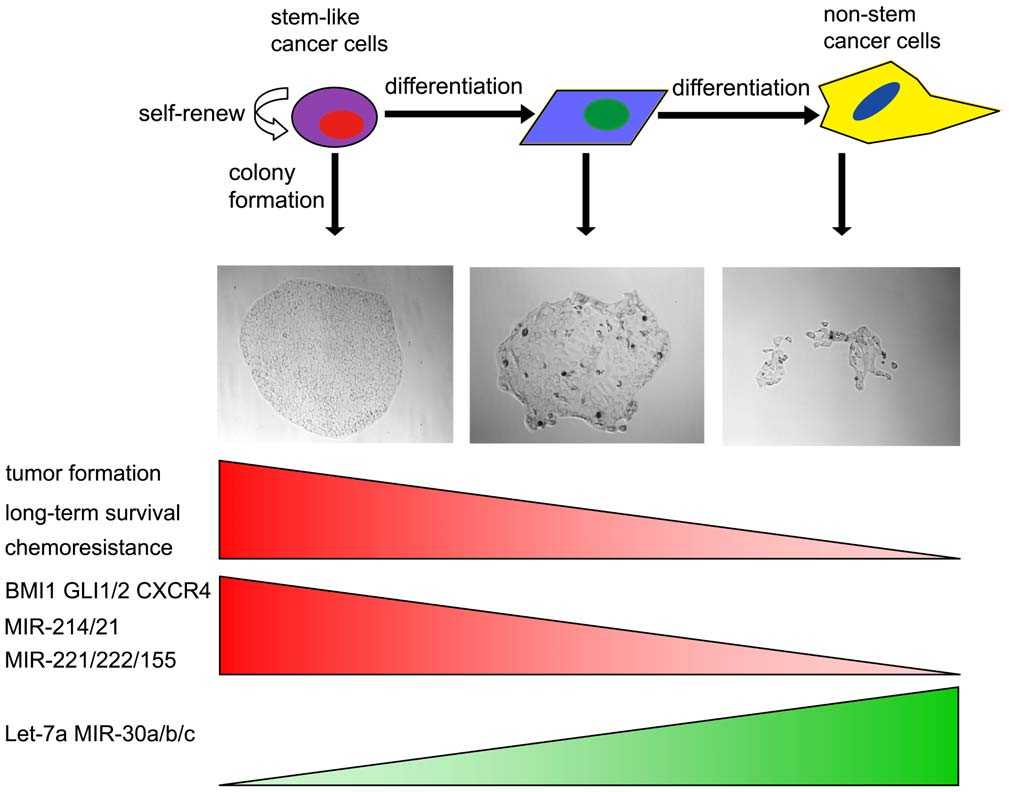
The model of hierarchical organization of pancreatic cancer cell population. The pancreatic cancer stem cells and their progenies in differentiation can develop to single colonies with different morphologies. Based on the in vivo and in vitro characteristics, Holoclones correspond to the cancer stem/progenitor cells, while paraclones correspond to the fully differentiated cells and meroclones correspond to the cells in the intermediate stage. The genes and microRNAs that associate with cancer stem cells and support chemoresistance, including *BMI1*, *GLI1/2*, *CXCR4*, *mir-21/214/221/222/155*, are enriched in holoclones. However, the *Let-7* and *mir-30a/b/c* are enriched in paraclones.

Taken together, the colonies with distinct morphologies and in different stages of differentiation can serve as a potential model for analysis of cancer stem cells (Fig. 7). With this model, genes and microRNAs potentially correlated with cancer stem cells can be identified. More importantly, parallel evaluation of chemotheraputic drugs can be carried out on cancer stem cells and autologous non-stem cancer cells. This means the clonal morphologies based cancer stem cell model will be useful to lead to the newer understanding of chemoresistance, which should be quite different from those obtained from heterogeneous cancer cell populations, and will be helpful to overcome the chemoresistance in cancer therapy.

## Materials and Methods

### Cell lines

Human pancreatic adenocarcinoma cell line BxPC3 (purchased from Cell Bank of China Academy of Sciences, Shanghai, China) and PC3 were (purchased from China Union Medical Collage, Beijing, China) cultured in RPMI-1640 (Hyclone) with 10% heat inactivated fetal bovine serum (Hyclone) and passaged as 1∶10 with 0.25% trypsin/EDTA (Hyclone) . These cell line were established from primary pancreatic ductal adenocarcinoma and with typical epithelium morphology [22], [23]. BxPC3 [22] was derived from European descent and PC3 [23] was derived from Chinese.

### Chemical drugs

Lyophilized powder of gemcitabine (Lilly) was dissolved in Calsium/Magnesium free PBS (Hyclone) and stored at −20°C with concentration of 4 mg/ml. 5-FU solution (Roche) was stored at −20°C with concentration of 25 mg/ml. Before use, stocks were diluted to working concentrations (5, 10, 50, 100, and 500 nM for 5-FU, and 1, 5, 10, 50, and 100 nM for Gemcitabine) with culture medium.

### Animals

All experiments were approved by the Animal Care and Use Committee of Peking University (approval number was IRB00001052-09051). NOD/SCID mice were purchased from Experimental Animal Sciences Center of Peking University and maintained in standard condition according to the institutional guidelines.

### Single cell cloning

Cells were harvested at 70%∼80% of confluence with Accumax (Chemicon) and resuspended in medium without serum. Single cell was seeded into each well of 96-well plates with MOFLO flow cytometry (DakoCytomation). Two days after plating, 96-well plates were checked with microscopy. Wells containing only one viable cell were marked. And then, medium was refreshed every 3 days. Colonies were classified as holo-, mero-, and paraclones according to their morphologies. 14 days after flow-cytometric sorting, typical colonies were selected for further experiments.

### Tumor cell implantation

Selected colonies were expanded and harvested with Accumax (Chemicon), counted and resuspended in 1∶1 mixture of RPMI-1640 and Matrigel (BD). Aliquots of cell suspension were injected subcutaneously into dorsolateral part of NOD/SCID mice. Tumor latency (i.e., time from injection to detection of palpable tumors) was determined. Within 9 weeks after implantation, tumor-bearing mice were sacrificed. Meanwhile, xenograft tumors were dissected out surgically and weighed. Mice with no sign of tumor burden were kept for at least 9 weeks since implantation and then examined on necroscopy to confirm that they were tumor-free.

For serial transplantation, xenograft tumors were minced into small pieces with scissors, suspended in M199 medium, and digested at 37°C for about 3 hours with 200units/ml ulturapure collagenas IV (Worthington Biochemicals). Further mechanical digestion was performed with a 25-ml pipette every 15 minutes. After digestion, cell suspension was filtered through a 40-µm nylon mesh and gently loaded onto the top layer of Histopaque-1077 gradient (Sigma-Aldrich) (1∼3×10^6^ cells/ml histopaque used in total volume of 3 mL) and then centrifuged at 400×g for 30 minutes at room temperature. Viable nucleated cells were collected at the interface, while red blood cells, dead cells and debris were eliminated. The harvested single-cell suspension was used for transplantation as described above.

### Chemoresistant assay

Cells from the same type of colonies were harvested, pooled together and seeded into 96-well plates with density of 5000 cells/well. 24 hours later, medium was refreshed and drugs (gemcitabine or 5-FU) were added. After 72 hours of drug treatment, 10% volume of WST-8 reagent (Beyotime) was added into all wells and plates were incubated at 37°C for 1 hour. The absorbance at 450 nm wavelength was measured with plate-reader (Bio-Rad). Based on the survival rate under different drug concentration, IC_50_ were calculated.

### Flowcytometric assay

Cells harvested from pooled colonies of the same type were resuspended in HBSS containing 2% FBS at concentration of 10^6^ cells/ml. Antibodies were then added into the sample aliquots. After 30 minutes incubation on ice, the samples were washed twice with HBSS with 2% FBS and analyzed with FACSCalibur (BD). Following antibodies were used: PE-Cy5 conjugated anti-human/mouse CD44 (eBioscience), PE conjugated anti-human CD24 (eBioscience), PE conjugated anti-human CD133 (miltenyibiotec) and APC conjugated anti-human CXCR4 (eBioscience). Pulse width and side scatter profiles were used to eliminate cell doublets, dead cells and cell debris.

### Quantitative RT-PCR

Total RNA (including microRNA) was extracted from the cells with miRNeasy Kit (Qiagen) according to the user manual. Reverse transcription reactions for both mRNAs and microRNAs were carried out with miScript Reverse Tanscription Kit (Qiagen).

Quantitative PCR assays for mRNAs were performed with SYBR Green PCR Master Mix (QPK-201) (TOYOBO). The PCR reactions were performed with following condition: 2 min at 95°C, followed by 35 rounds of 15 sec at 95°C and 1 min at 60°C. The relative expression of each gene was normalized against GAPDH. Primers used were shown in Table S1.

Quantitative PCR assays for microRNAs were performed with the miScript SYBR® Green PCR Kit (Qiagen). The PCR reactions were performed with following condition: 15 min at 95°C, followed by 35 rounds of 15 sec at 95°C and 30 sec at 55°C and 30 sec at 72°C. The relative expression of each microRNA was normalized against RNU-6b. Primers were all purchased from Qiagen. Their codes and catalog numbers were shown in Table S2.

All assays were performed with ABI PRISM 7300 Sequence Detection System and under the control of ABI 7300 SDS Software version 1.3.1.

### H&E staining

Tissue samples were dissected from mice and fixed in 10% phosphate buffered formalin. After fixed, tissues were embedded in paraffin. According to standard histopathologic procedures, sections were cut 4-Am thick, mounted on poly-L-lysine coated slides, dried overnight at 37°C, dewaxed in xylene, rehydrated and stained with H&E.

## Supporting Information

Figure S1 **Colony heterogeneity in pancreatic cancer cell line PC3.** Panels show representative holoclones (A), meroclones (B) and paraclones (C) from PC3 cultures. All photographs were taken at 2 weeks after plating (Bar, 100 microns). At this time point, each type of colonies was counted (D). The results from repeated experiments (n = 4) are presented as means± s.e.m in histogram.
(TIF)

Figure S2 **Self-renewal capacity of distinct types of colonies derived from PC3 cell line.** Cells isolated from single colonies were plated at low density under common condition. (A) At the initial passage, holoclones (n = 9) mainly produced similar frequencies of descendant holoclones and meroclones, whereas much lower percentages of paraclones were generated. (B)After passages of one month, holoclones (n = 8) generated the full range of progeny colonies with the frequencies similar to those retained in unsorted parental PC3 cell line. (C) Meroclones (n = 8) mainly produced similar amount of paraclones and meroclones, and rare holoclones were generated simultaneously.
(TIF)

Figure S3 **Long-term propagation capacity of distinct types of colonies derived from PC3 cell line.** Holoclones (n = 9, red line), meroclones (n = 8, green line) and paraclones (n = 8, blue line) were passage under common condition for 2 months. Life spans of each colony were recorded. 4 of 8 meroclones and 6 of 8 paraclones were aborted gradually, whereas 8 of 9 holoclones remained viable (p<0.01).
(TIF)

Figure S4 **Histological characteristics of exnograft tumors derived from unsorted BxPC3 cell line and holoclones.** 10^4^ cells from unsorted cell line and holoclones were employed to produce xenograft tumors. H&E staining was performed to analyze the histological features of xenograft tumors derived from unsorted BxPC3 cell line (A) and holoclones (C) (Bar, 400 microns). The selected areas (black box) in panel A and C were magnified as panel B and D respectively (Bar, 100 microns).
(TIF)

Figure S5 **Expression of cancer stem cell markers CD44 and CD24 in holoclones and paraclones derived from pancreatic cancer cell line PC3.** Cells in holoclones and paraclones derived from PC3 cell line were examined with flowcytometry for the cell surface markers of cancer stem cells. Flowcytometric plots (A) showed that CD44 (trunnion axis) was strongly positive and with little difference among distinct types of colonies. Three gates were set up to show the high (R1), medium (R2) and low (R3) level of CD24 (vertical axis) expression. Representative plots showed that cells isolated from paraclones (right panel) tended to be distributed in gate R1 while cells isolated from holoclones (left panel) were dominantly distributed in gate R3. The distribution of cells in three gates was summarized (B) as below (p<0.05).
(TIF)

Table S1 Primers for real-time PCR of genes.
(DOC)

Table S2 Primers for real-time PCR of microRNAs.
(DOC)

Table S3 Tumorgenecity of distinct types of colonies derived from PC3 cell line.
(DOC)
