# Supplementary figures and images for: Holoclone Forming Cells from Pancreatic Cancer Cells Enrich Tumor Initiating Cells and Represent a Novel Model for Study of Cancer Stem Cells
Source: PLoS One. 2011 Aug 3;6(8):e23383. doi: 10.1371/journal.pone.0023383 (PMC3149653; doi:10.1371/journal.pone.0023383)

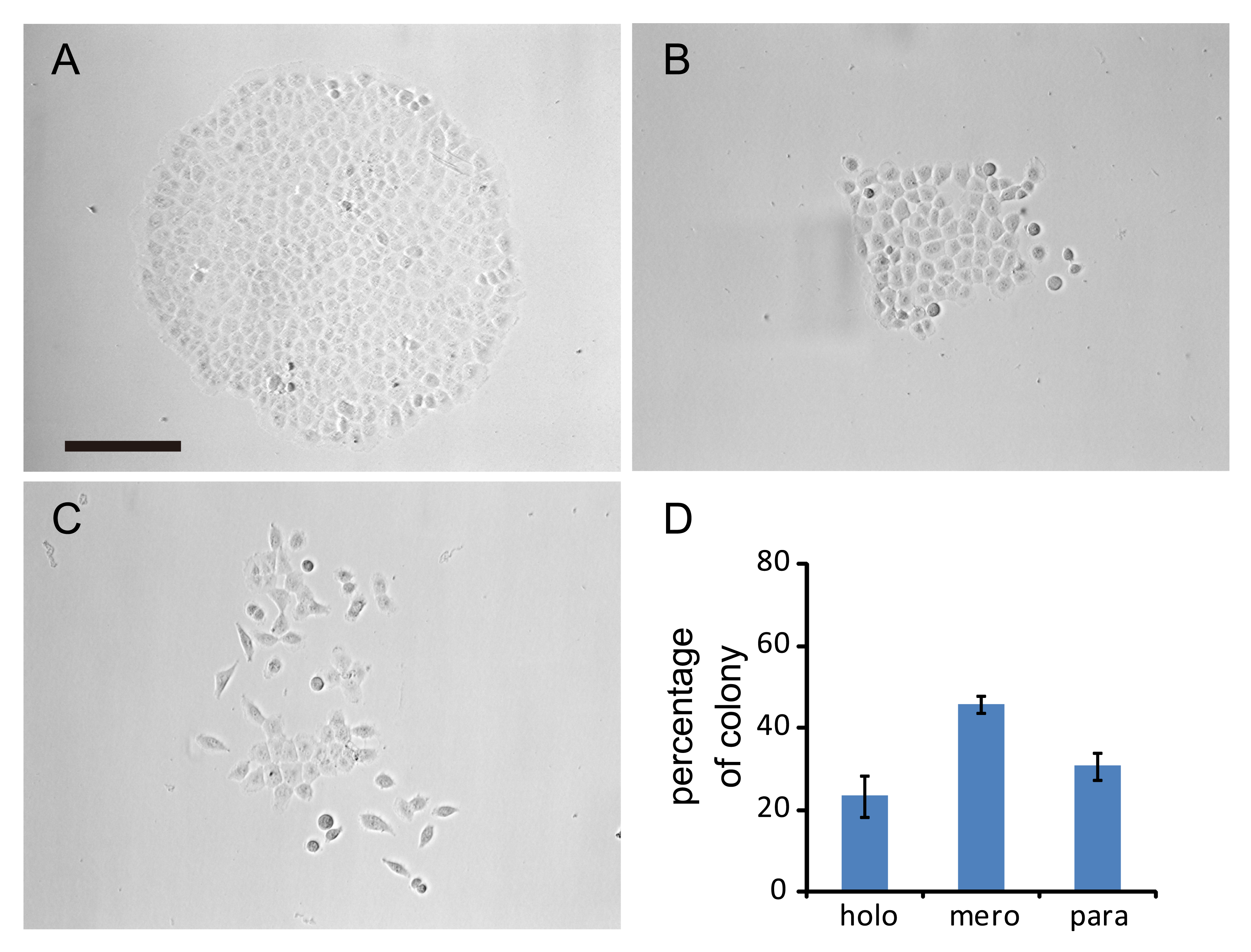

Supplement: Figure S1 — Colony heterogeneity in pancreatic cancer cell line PC3. Panels show representative holoclones (A), meroclones (B) and paraclones (C) from PC3 cultures. All photographs were taken at 2 weeks after plating (Bar, 100 microns). At this time point, each type of colonies was counted (D). The results from repeated experiments (n = 4) are presented as means± s.e.m in histogram. (TIF) [file pone.0023383.s001.tif]

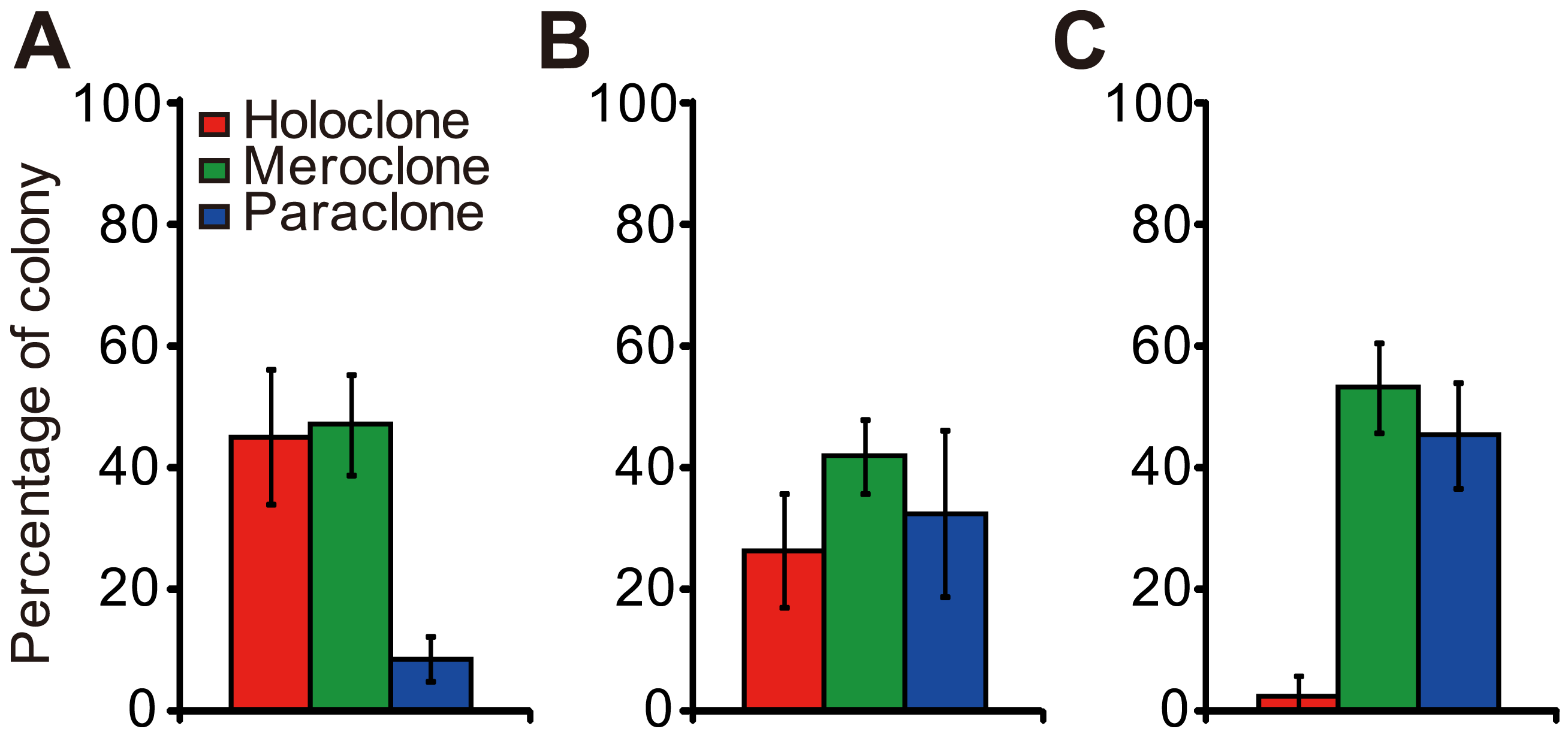

Supplement: Figure S2 — Self-renewal capacity of distinct types of colonies derived from PC3 cell line. Cells isolated from single colonies were plated at low density under common condition. (A) At the initial passage, holoclones (n = 9) mainly produced similar frequencies of descendant holoclones and meroclones, whereas much lower percentages of paraclones were generated. (B)After passages of one month, holoclones (n = 8) generated the full range of progeny colonies with the frequencies similar to those retained in unsorted parental PC3 cell line. (C) Meroclones (n = 8) mainly produced similar amount of paraclones and meroclones, and rare holoclones were generated simultaneously. (TIF) [file pone.0023383.s002.tif]

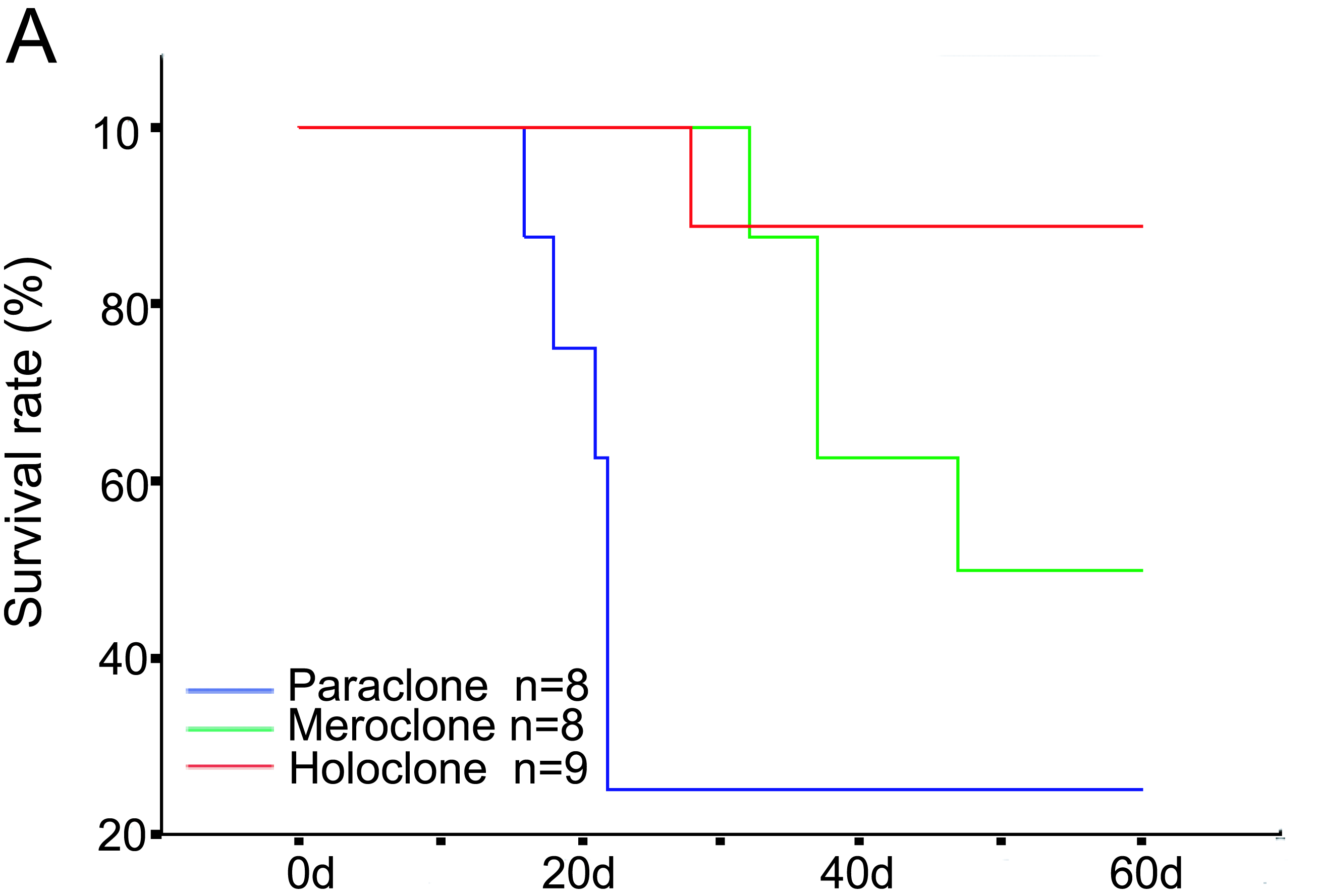

Supplement: Figure S3 — Long-term propagation capacity of distinct types of colonies derived from PC3 cell line. Holoclones (n = 9, red line), meroclones (n = 8, green line) and paraclones (n = 8, blue line) were passage under common condition for 2 months. Life spans of each colony were recorded. 4 of 8 meroclones and 6 of 8 paraclones were aborted gradually, whereas 8 of 9 holoclones remained viable (p<0.01). (TIF) [file pone.0023383.s003.tif]

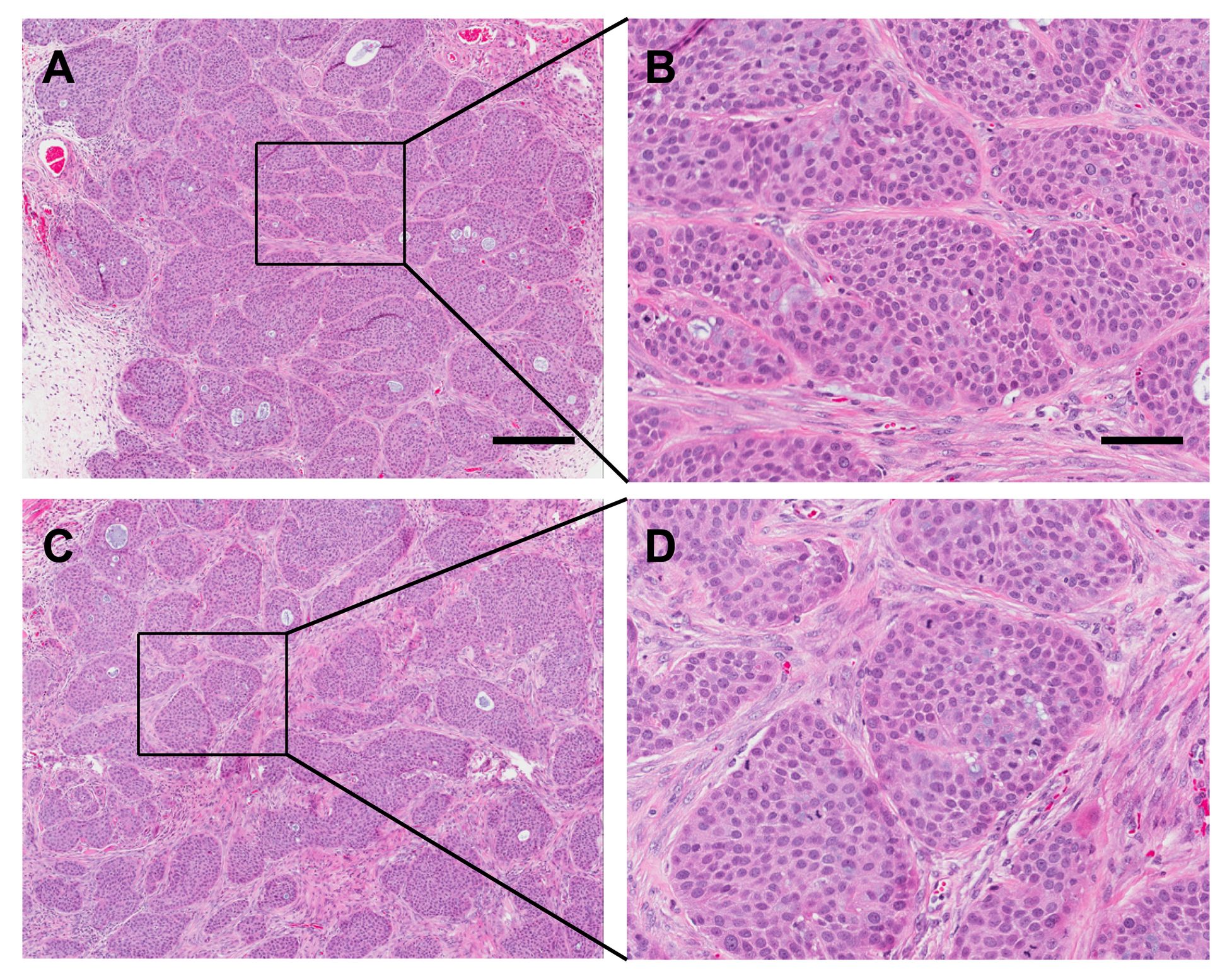

Supplement: Figure S4 — Histological characteristics of exnograft tumors derived from unsorted BxPC3 cell line and holoclones. 104 cells from unsorted cell line and holoclones were employed to produce xenograft tumors. H&E staining was performed to analyze the histological features of xenograft tumors derived from unsorted BxPC3 cell line (A) and holoclones (C) (Bar, 400 microns). The selected areas (black box) in panel A and C were magnified as panel B and D respectively (Bar, 100 microns). (TIF) [file pone.0023383.s004.tif]

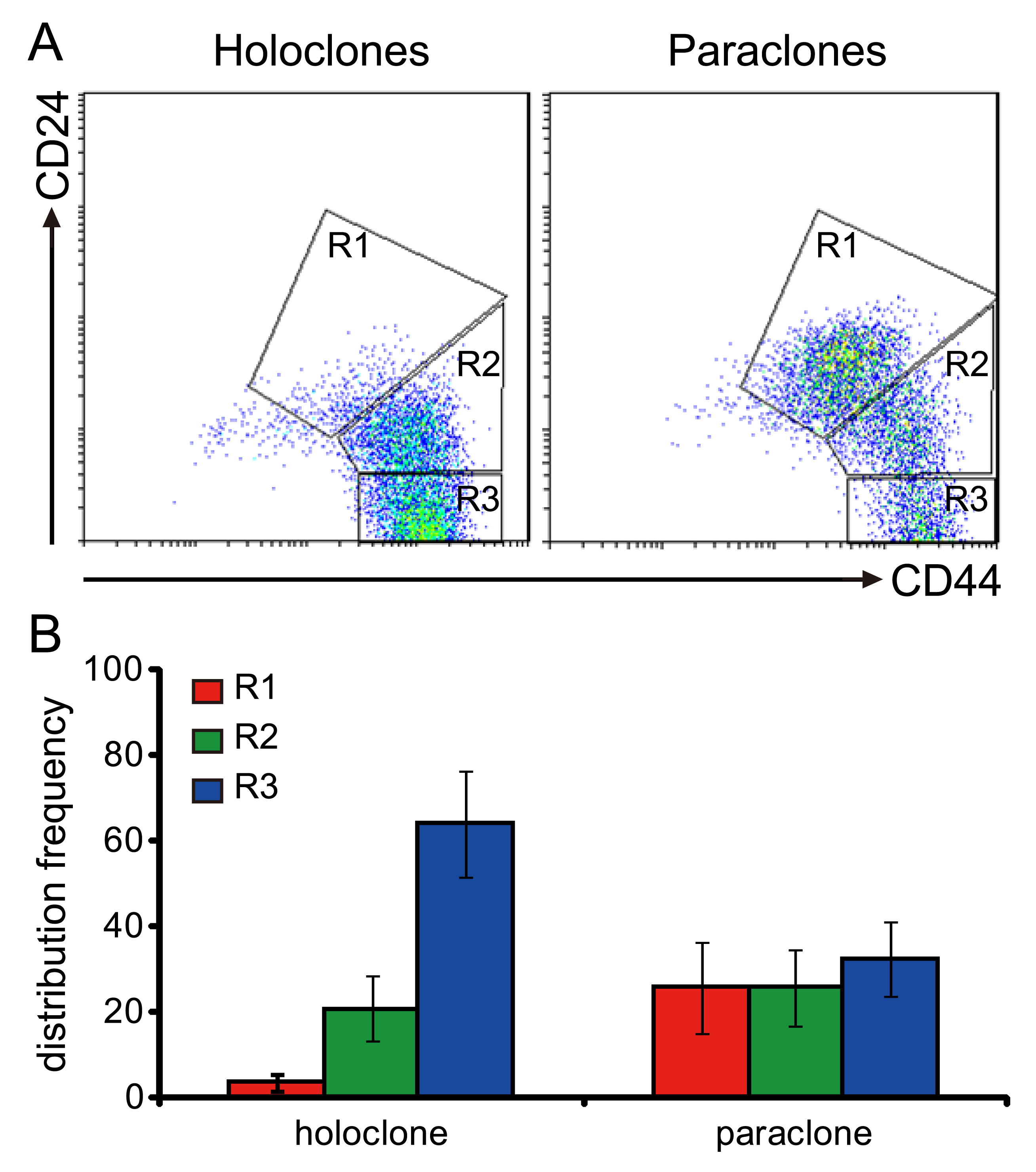

Supplement: Figure S5 — Expression of cancer stem cell markers CD44 and CD24 in holoclones and paraclones derived from pancreatic cancer cell line PC3. Cells in holoclones and paraclones derived from PC3 cell line were examined with flowcytometry for the cell surface markers of cancer stem cells. Flowcytometric plots (A) showed that CD44 (trunnion axis) was strongly positive and with little difference among distinct types of colonies. Three gates were set up to show the high (R1), medium (R2) and low (R3) level of CD24 (vertical axis) expression. Representative plots showed that cells isolated from paraclones (right panel) tended to be distributed in gate R1 while cells isolated from holoclones (left panel) were dominantly distributed in gate R3. The distribution of cells in three gates was summarized (B) as below (p<0.05). (TIF) [file pone.0023383.s005.tif]
