# Supplementary material for: Holoclone Forming Cells from Pancreatic Cancer Cells Enrich Tumor Initiating Cells and Represent a Novel Model for Study of Cancer Stem Cells
Source: PLoS One. 2011 Aug 3;6(8):e23383. doi: 10.1371/journal.pone.0023383 (PMC3149653; doi:10.1371/journal.pone.0023383)
Supplement: Table S1 — Primers for real-time PCR of genes. (DOC) [file pone.0023383.s006.doc]

**Table S1. Primers for real-time PCR of genes**

| Gene name | Forward primer (5'to3') | Reverse primer (5'to3') |
| --- | --- | --- |
| SLC28A1 | TGGAGCGCTGGAAATCTCAG | GAGCTGCCTGGTGAAATGGT |
| SLC28A2 | CGTCTGTGCCTAAGGCTGCT | TGAGTTGTGGGCACCCTGAG |
| SLC28A3 | CGGACTCACATCCATGGCTC | TGCAGGCTGTCATGAAGCAG |
| SLC29A1 | CGCCTGGAATTCTACCGCT | GCTCTTGGCTCCTCTCCTTTG |
| SLC29A2 | TCACCGAAGCCTAATTGAGGC | GACCAGTCACTTTCCCCAGG |
| SLC29A3 | GACATTGGTGCTTCAGAGCCT | TCTGCTCTCTGTCCCCAAGTG |
| CD44 | TGACTAAATCAGGGCTGGGC | GAGGAGCAGAGGCTGGGAAT |
| CD24 | TGCTCCTACCCACGCAGATT | GCCTTGGTGGTGGCATTAGT |
| CXCR4 | GCATGACGGACAAGTACAGGC | CAAAGTACCAGTTTGCCACGG |
| GLI1 | CGGAAGTCATACTCACGCCTC | GAAGGCTTTACTGCAGCCCTC |
| GLI2 | CTGTCGCCATTCACAAGCG | CACATGAGCCGTGTCCAGAC |
| GLI3 | GCGCCAAACACCAAAACAG | TGTTTCCGGAGGGAGCTTG |
| BMI1 | TGGAAAGTGACTCTGGGAGTGAC | GATGAGGAGACTGCACTGGAGTAC |
| GAPDH | TGCACCACCAACTGCTTAGC | GGCATGGACTGTGGTCATGAG |
