# Supplementary material for: Holoclone Forming Cells from Pancreatic Cancer Cells Enrich Tumor Initiating Cells and Represent a Novel Model for Study of Cancer Stem Cells
Source: PLoS One. 2011 Aug 3;6(8):e23383. doi: 10.1371/journal.pone.0023383 (PMC3149653; doi:10.1371/journal.pone.0023383)
Supplement: Table S2 — Primers for real-time PCR of microRNAs. (DOC) [file pone.0023383.s007.doc]

**Table S2. Primers for real-time PCR of microRNAs**

| microRNA | Code of primer | Catalog number |
| --- | --- | --- |
| Let-7a | Hs_let-7a_1 | MS00006482 |
| miR-155 | Hs_miR-155_1 | MS00003605 |
| miR-222 | Hs_miR-222_2 | MS00007609 |
| miR-214 | Hs_miR-214_1 | MS00003822 |
| miR-21 | Hs_miR-21_2 | MS00009079 |
| miR-221 | Hs_miR-221_1 | MS00003857 |
| miR-30a | Hs_miR-30a-5p_1 | MS00007350 |
| miR-30b | Hs_miR-30b_1 | MS00003276 |
| miR-30c | Hs_miR-30c_2 | MS00009366 |
| RNU-6b | Hs_RNU6B_2 | MS00014000 |
