# Supplementary material for: Holoclone Forming Cells from Pancreatic Cancer Cells Enrich Tumor Initiating Cells and Represent a Novel Model for Study of Cancer Stem Cells
Source: PLoS One. 2011 Aug 3;6(8):e23383. doi: 10.1371/journal.pone.0023383 (PMC3149653; doi:10.1371/journal.pone.0023383)
Supplement: Table S3 — Tumorgenecity of distinct types of colonies derived from PC3 cell line. (DOC) [file pone.0023383.s008.doc]

**Table S3. Tumorgenecity of distinct types of colonies derived from PC3 cell line.**

| Types of colonies | Clone | Cell number | Tumor incidence | Mean weight of tumor (g) | Termination/latency（day） |
| --- | --- | --- | --- | --- | --- |
| Unsorted |  | 10^6^ | 100% (3/3) |  | 30/14 |
|  |  | 10^5^ | 100% (3/3) |  | 30/14 |
|  |  | 10^4^ | 100% (6/6) |  | 30/21 |
|  |  | 10^3^ | 100% (6/6) |  | 30/21 |
| Para | G11 | 10^4^ | 0%（0/6） |  | 63 |
|  | G11 | 10^5^ | 0%（0/6） |  | 63 |
| Mero | C07 | 10^5^ | 0%（0/6） |  | 63 |
|  | C07 | 10^6^ | 0%（0/6） |  | 63 |
|  | C02 | 10^5^ | 0%（0/6） |  | 63 |
|  | C02 | 10^6^ | 0%（0/6） |  | 63 |
|  | E02 | 10^5^ | 0%（0/6） |  | 63 |
|  | E02 | 10^6^ | 0%（0/6） |  | 63 |
| Holo | C06 1º | 10^4^ | 100%（6/6） | 0.51 | 65/18 |
|  | C06 2º | 10^4^ | 100%（6/6） | 0.28 | 36/17 |
|  | G03 1º | 10^4^ | 100%（6/6） | 0.4 | 65/16 |
|  | G03 2º | 10^4^ | 100%（6/6） | 0.34 | 36/18 |
|  | G11 1º | 10^4^ | 100%（6/6） | 0.4 | 65/15 |
|  | G11 2º | 10^4^ | 100%（6/6） | 0.19 | 36/17 |

NOTE: Cells from PC3 clones (1º) at the indicated numbers were injected subcutaneously with 1﹕1 mixture of RPMI-1640 and Matrigel into the dorsolateral part of NOD/SCID mice. Secondary (2º) transplantation experiments were carried out as described in “materials and methods”.
